# Targeting T-cell malignancies using anti-CD4 CAR NK-92 cells

**DOI:** 10.18632/oncotarget.22626

**Published:** 2017-11-22

**Authors:** Kevin G. Pinz, Elizabeth Yakaboski, Alexander Jares, Hua Liu, Amelia E. Firor, Kevin H. Chen, Masayuki Wada, Huda Salman, William Tse, Nabil Hagag, Fengshuo Lan, Elaine Lai-Han Leung, Xun Jiang, Yupo Ma

**Affiliations:** ^1^ iCell Gene Therapeutics LLC, Research & Development Division, Long Island High Technology Incubator, Stony Brook, NY 11790, USA; ^2^ State Key Laboratory of Quality Research in Chinese Medicine, Macau Institute for Applied Research in Medicine and Health, Macau University of Science and Technology, Macau SAR, China; ^3^ Department of Pathology, Stony Brook Medicine, Stony Brook University Medical Center, Stony Brook, NY 11794, USA; ^4^ Department of Internal Medicine, Stony Brook Medicine, Stony Brook University Medical Center, Stony Brook, NY 11794, USA; ^5^ Division of Hematology and Medical Oncology, James Graham Brown Cancer Center, University of Louisville Health Sciences Center, Louisville, KY 40202, USA

**Keywords:** NK cells, immunotherapy, T-cell malignancies, chimeric antigen receptors

## Abstract

Peripheral T-cell lymphomas (PTCLs) are a group of very aggressive non-Hodgkin's lymphomas (NHLs) with poor prognoses and account for a majority of T-cell malignancies. Overall, the standard of care for patients with T-cell malignancies is poorly established, and there is an urgent clinical need for a new approach. As demonstrated in B-cell malignancies, chimeric antigen receptor (CAR) immunotherapy provides great hope as a curative treatment regimen. Because PTCLs develop from mature T-cells, these NHLs are commonly CD4^+^, and CD4 is highly and uniformly expressed. Therefore, CD4 is an ideal target for PTCL CAR immunotherapy. To that effect, we created a robust third-generation anti-CD4 CAR construct (CD4CAR) and introduced it into clonal NK cells (NK-92). CD4CAR NK-92 cells specifically and robustly eliminated diverse CD4^+^ human T-cell leukemia and lymphoma cell lines (KARPAS-299, CCRF-CEM, and HL60) and patient samples *ex vivo*. Furthermore, CD4CAR NK-92 cells effectively targeted KARPAS-299 cells *in vivo* that modeled difficult-to-access lymphoma nodules, significantly prolonging survival. In our study, we present novel targeting of CD4 using CAR-modified NK cells, and demonstrate efficacy. Combined, our data support CD4CAR NK cell immunotherapy as a potential new avenue for the treatment of PTCLs and CD4^+^ T-cell malignancies.

## INTRODUCTION

Peripheral T-cell Lymphomas (PTCLs) are typically aggressive lymphomas that develop from mature T cells [1]. Most T-cell malignancies are PTCLs, which account overall for 10–15% of all non-Hodgkin lymphomas (NHLs). Compared to B-cell NHLs, PTCLs are more difficult to treat, have poorer outcomes and lower median survival [1–4]. Indeed, following chemotherapy/medical treatment, most PTCL patients present with relapsed, refractory or minimal residual disease, and are therefore not eligible for hematopoietic stem cell transplantation, currently the only potentially curative option [1, 3, 5]. As the standard of care is not well-established, PTCLs constitute an urgent unmet clinical need [1, 5, 6]. Therefore, chimeric antigen receptor (CAR) therapy is a promising strategy for the treatment of PTCLs. To date, most chimeric antigen receptor (CAR) hematological immunotherapy has been directed against B-cell malignancies, with many instances of complete response, however reports of CAR targeting T-cell malignancies are increasing [7–12].

Because they develop from T cells, PTCLs are commonly CD4^+^, and the CD4 expression is often high and consistent making it an ideal target for PTCL CAR therapy. Additionally, malignant CD4 expression is not limited to T-cell malignancies, and can be expressed aberrantly in other hematological malignancies [13]. Therefore robust validation of a CAR therapy for PTCLs would entail effective and CD4-specific eradication.

CAR technology redirects cytotoxic immune cells specifically against a surface antigen in a major-histocompatibility complex (MHC)-independent manner. The CAR extracellular single-chain variable fragment (scFv) is specific for the target antigen and is linked by hinge and transmembrane regions to CD28 and/or 4-1BB co-activation domains and finally the CD3ζ intracellular signaling domain [14]. Although the ideal CAR target is expressed on 100% of tumor cells with 100% specificity, tumor-associated antigens are usually expressed on only a subset of cancer cells, unless the normal cells from which the tumor is derived also share that antigen, as is the case with CD4. However, anti-CD4 targeting protects non-hematological tissue, as CD4 expression is restricted to the hematopoietic compartment. Crucially, stem cell rescue is available for hematological immunotherapy. Previously, anti-CD4 antibodies have been used in non-human primate models [15] as well as in clinical trials for cutaneous and peripheral T cell lymphoma [16–19]. Overall, short-term CD4^+^ cell depletion is well-tolerated, reversible, and often no clinical evidence of immunosuppression is detected [16].

In a previous study, we demonstrated that CD8^+^ T cells could be redirected against CD4^+^ T-cell malignancies using anti-CD4 CAR [20]. Subsequently, we used that study for the successful application and subsequent designation of FDA Orphan Drug status for PTCLs using CD4CAR T cells. Nevertheless, since T cells can persist and engraft for several months, this raises potential concern regarding prolonged CD4^+^ cell aplasia by CAR therapy, which can result in deleterious effects not limited to opportunistic infections and viral reactivations. If used as allogeneic therapy, CAR T-cells need to be HLA-matched, with risk of graft-versus host disease. An alternative strategy employing NK cells could potentially address these concerns for translation into the clinic.

Natural killer (NK) cells mediate anti-tumor effects without the risk of GvHD and are short-lived relative to T-cells, with a turnover time of around 2 weeks [21]. CAR NK cells would be exhausted shortly after lysing target cells [22], however the inclusion of an inducible suicide switch would provide an additional safeguard to ablate the immunotherapy should it persist beyond the therapeutic window [23, 24]. NK cells are uniformly CD56^+^, CD3^-^ and are part of both the innate and adaptive immune responses, with a distinct lineage origin in hematopoiesis [25]. Previous pre-clinical studies have redirected CAR-modified primary human NK cells against CD19 [26, 27], CD20 [28], CD244 [29], and HER2 [30], and anti-CD19 CAR-modified donor-derived and haploidentical NK cells have successfully entered clinical trials for B-cell acute lymphoblastic leukemia (NCT00995137 and NCT01974479). Additionally, NK-92, a human NK cell line, has been used in a variety of clinical studies for solid tumor and hematologic malignancies [31, 32], and also in pre-clinical CAR applications [33–39], and is therefore not only a good model for clonal NK cells, but also for autologous or allogeneic NK cell immunotherapy.

Here, we engineered NK-92 cells to express a third generation CD4-specific CAR (CD4CAR) containing CD28, 4-1BB and CD3ζ signaling domains, because third-generation CAR constructs have been associated with enhanced antitumor activity [40, 41]. We found that CD4CAR NK-92 cells exhibit robust anti-tumor cytotoxicity *ex vivo* against both adult and pediatric CD4^+^ lymphoma/leukemia cell lines, CD4^+^ T-cells isolated from umbilical cord blood, as well as against untreatable primary CD4^+^ T-cell malignancies from adult and pediatric patients. CD4CAR NK-92 cells also present potent *in vivo* anti-CD4 activity in xenogeneic mouse models. Consistent with CD4 as a mature T-cell marker, CD4CAR NK-92 cells did not significantly affect CD34+ cord blood granulocyte/macrophage or erythroid colony formation (CFU) *ex vivo*. Therefore, CD4CAR NK-92 cells did not affect the hematopoietic stem cell and progenitor compartment *ex vivo*, suggesting preserved stem cell and progenitor capacity to repopulate. Together, these pre-clinical efficacy and specificity data support potential use of CD4CAR NK cells as a conditioning regimen component, as part of a bridge-to-transplant strategy which would allow patients with no therapeutic options to qualify for curative hematopoietic stem cell transplantation, or as a stand-alone curative treatment modality without transplant.

## RESULTS

### Generation of the third generation CD4CAR

**Figure 1:**
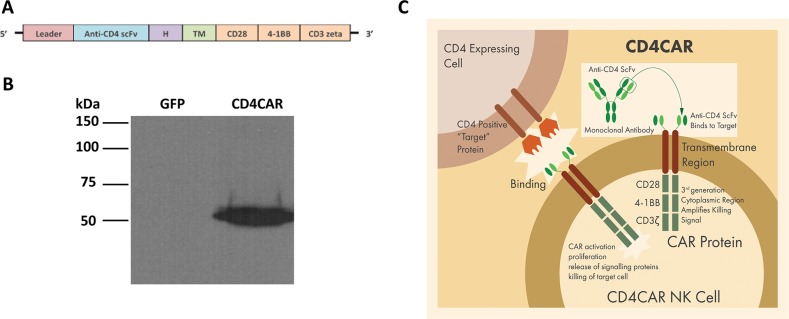
CD4CAR construct **(A)** Schematic representation of recombinant lentiviral vector encoding third generation CD4CAR, driven by spleen focus-forming virus (SFFV) promoter. The construct contains a leader sequence, anti-CD4 scFv, hinge domain (H), transmembrane (TM) and signaling domains CD28, 4-1BB, and CD3 zeta. **(B)** HEK293FT cells were transfected with GFP vector control (lane 1) and CD4CAR (lane 2) lentiviral plasmids. Forty-eight hours after transfection, cells were removed and subsequently used for Western blot analysis with mouse anti-human CD3z antibody. **(C)** Illustration of third-generation CAR NK-92 cells targeting CD4 expressing cells.

The single-chain variable fragment (scFv) nucleotide sequence of the anti-CD4 molecule was derived from the humanized monoclonal antibody (mAb) ibalizumab (Hu5A8 or TNX-355)− a well-studied and characterized mAb with well-defined and avid binding capability previously used in clinical trials for HIV [42, 43]. To improve signal transduction, the CD4CAR was designed with CD28 and 4-1BB domains fused to the CD3zeta signaling domain, to create a third generation CAR. CD19-targeting third generation CAR T-cells have previously been used in clinical trials, with astounding efficacy [44]. For efficient expression of the CD4CAR molecule on the NK-92 cell surface, a strong spleen focus-forming virus promoter (SFFV) was used and the leader sequence of CD8 was incorporated in the construct. The anti-CD4 scFv was separated from the intracellular signaling domains by CD8-derived hinge (H) and transmembrane (TM) regions (Figure 1A and 1C). The CD4CAR DNA molecule was subsequently sub-cloned into a lentiviral plasmid.

### Characterization of CD4CAR

In order to validate the CD4CAR construct, HEK293-FT cells were transfected with the CD4CAR lentiviral plasmid or vector control plasmid, and 48 hours later were harvested for Western blot analysis. Immunoblotting with an anti-CD3zeta monoclonal antibody showed bands of predicted size for the CD4CAR-CD3zeta fusion protein (Figure 1B). As expected, no CD3zeta expression was observed for the GFP vector control protein (Figure 1B).

### Generation of CD4CAR NK-92 cells

**Figure 2:**
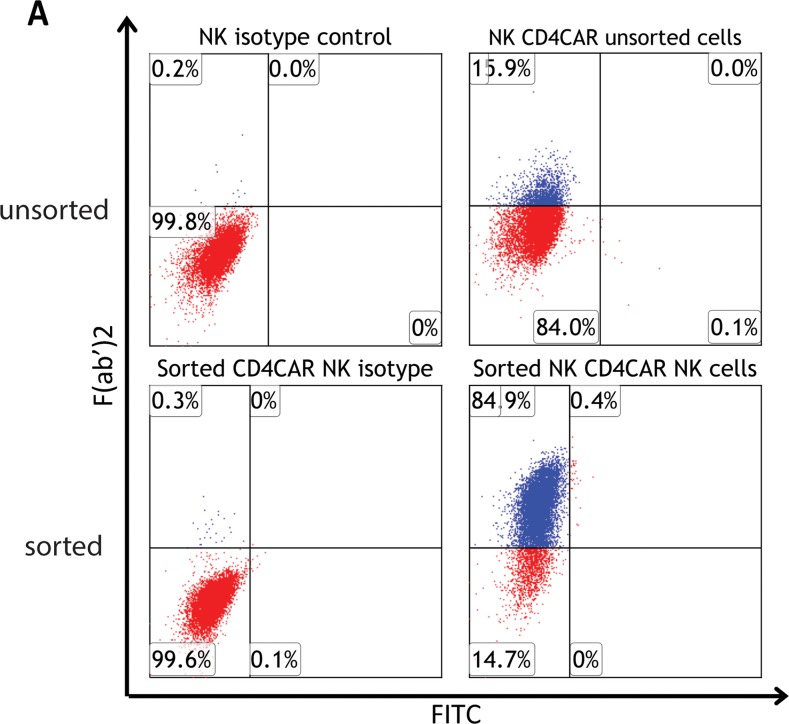
CD4CAR NK-92 cell production (**A**, upper panel) CD4CAR expression levels on NK-92 cells prior to being sorted by FACS (N=3); (**A**, lower panel) CD4CAR expression on NK-92 cells after sorting and expansion, prior to co-culture experiments (N=3).

CD4CAR NK-92 transduction efficiency was determined to be 15.9%, as determined by flow cytometry (Figure 2A upper panel). Next, fluorescence-activated cell sorting (FACS) was used to further enrich for CD4CAR^+^ NK-92 cells. Following sorting, collected CD4CAR^high^ NK-92 cells were confirmed to be more than 85% CD4CAR positive (Supplementary Figure 2). After FACS collection of CD4CAR^high^ cells, CD4CAR expression levels remained consistently stable at 75-90% on NK -92 cells during expansion of up to 10 passages, and following cryopreservation. Indeed, at the onset of co-culture experiments, expanded CD4CAR^high^ NK-92 cells expressed CAR at 85% (Figure 2A, lower panel).

### CD4CAR NK-92 cells specifically lyse CD4^+^ tumor cells

CD4CAR NK-92 cells were tested *ex vivo* for anti-CD4 activity using the following CD4^+^ cell lines: KARPAS-299, HL-60, and CCRF-CEM. The KARPAS-299 cell line is a PTCL established from the peripheral blood of a 25-year-old patient with anaplastic large T-cell lymphoma. The HL-60 cell line was established from the peripheral blood of a 36-year-old patient with acute promyelocytic leukemia with aberrant CD4 expression. Finally, the CCRF-CEM cell line was established from the peripheral blood of a 4-year-old patient with T-cell acute lymphoblastic leukemia (T-ALL).

**Figure 3:**
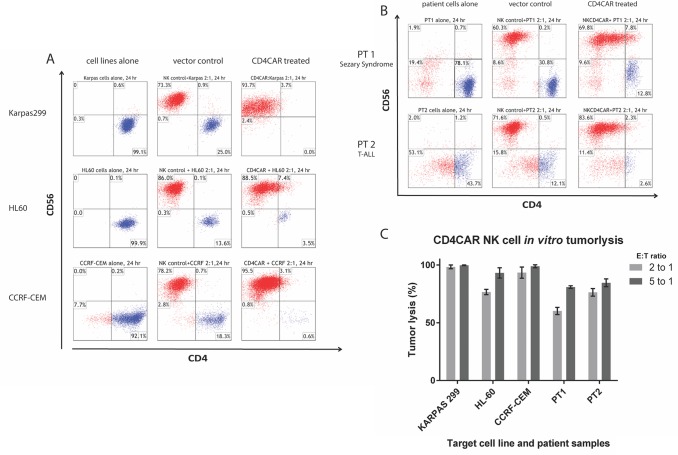
CD4CAR NK-92 cells ablate CD4+ leukemia and lymphoma cells in co-culture assays Co-culture experiments were performed at an effector to target ratio of 2:1 for 24 hours and were directly analyzed by flow cytometry for CD56 and CD4 (panels A and B). Each assay consists of target cells alone control (left), and target cells incubated with NK-92 cells transduced with vector control (center) or CD4CAR (right) lentiviral supernatant. **(A)** Top row: KARPAS-299 (N=3). Middle row: HL-60 T-cells (N=2). Bottom row: CCRF-CEM cells (N=2). **(B)** CD4CAR NK-92 cells eliminated primary T-cell leukemia cells from a patient with CD4^+^ T-cell lymphoma/ Sézary syndrome (N=2) and CD4 expressing pediatric T-cell ALL (N=2). **(C)** Bar graph summarizing co-culture assay results for both 2:1 and 5:1 E:T ratios.

**Figure 5:**
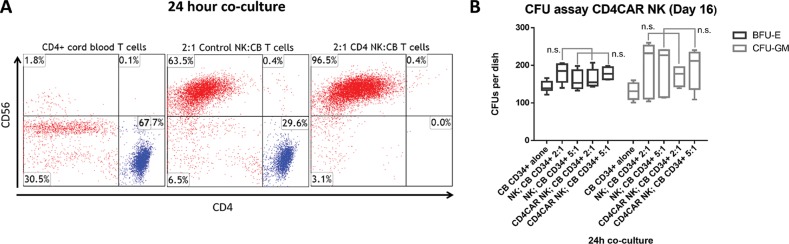
CD4CAR NK-92 cells eliminate CD4+ T-cells isolated from human cord blood at an effector to target ratio of 2:1, but do not affect hematopoietic stem cell/progenitor compartment output **(A)** Co-culture assays were performed at an effector to target ratio of 2:1 for 24 hours, after which, cells were stained with mouse anti-human CD56 and CD4 antibodies. Target cells were incubated alone as a control (left). NK-92 cells were transduced with either vector control (center) or CD4CAR (right) lentiviral supernatant and incubated with CD4^+^ T-cells obtained from human cord blood. (N=2) **(B)** CD4CAR NK-92 cells were incubated at co-culture effector:target ratios of 2:1 and 5:1 respectively with 500 CD34+ cord blood cells for 24 hours in NK cell media supplemented with IL-2. Experimental controls used were CD34+ cells alone, and non-transduced NK-92 cells were co-cultured at respective 2:1 and 5:1 effector:target ratios with CD34+ CB cells. Hematopoietic compartment output was assessed via formation of erythroid burst-forming units (BFU-E) and number of granulocyte/monocyte colony-forming units (CFU-GM) at Day 16. CFU statistical analysis was performed via 2-way ANOVA with alpha set at 0.05.

During 24-hour co-culture experiments, CD4CAR NK-92 cells showed profound killing of CD4^+^ leukemia/lymphoma cells at the low effector cell to target cell ratio (E:T) of 2:1 (Figure 3A) and the standard 5:1 ratio (Figure 3C and Supplementary Figure 1). In order to demonstrate robustness and rigor we present 2:1 E:T ratio replicates (Figures 3, 5) for corresponding 5:1 E:T ratio replicates (Supplementary Figure 1). In co-culture cytotoxicity assays, target tumor cells were identified by the CD4^+^, CD56^-^ immunophenotype (labeled in blue on flow cytometry charts).

Strikingly, at a low E:T ratio of 2:1, CD4CAR NK-92 cells completely ablated 100% of KARPAS-299 cells compared to vector control (N=2) (Figure 3B upper panel and 3c). Similarly, at a low E:T ratio of 2:1, CD4CAR NK-92 cells robustly lysed 75% of HL-60 cells and 97% of CCRF-CEM cells, as compared to vector control (Figure 3A and 3C). Combined, these data across several CD4^+^ tumor cells lines demonstrate that CD4CAR NK-92 cells potently target CD4^+^ leukemic cells, in a specific and reliable manner. It is important to note that static cytotoxicity *ex vivo* assays do not fully recapitulate the human microenvironment and thus severely underestimate actual potency in the clinic, and that these data compare favorably to analogous CAR studies in terms of percentage tumorlysis [14, 15, 17].

Co-culture studies were also conducted using patient samples (Figures 3B and 3C). Patient 1 presented with Sézary syndrome, an aggressive form of CD4^+^ cutaneous T-cell lymphoma unresponsive to standard chemotherapy. Sézary syndrome is a subset of PTCL (Figure 3B). Patient 2 presented with a CD4^+^ pediatric T-cell acute lymphoblastic leukemia (T-ALL) (Figure 3B). After 24 hours of co-culture at a low E:T ratio of 2:1, CD4CAR NK-92 cells eradicated 58% of CD4^+^ Sézary syndrome cells from patient 1, and 78% of CD4^+^ T-ALL cells from patient 2 (N=2). Furthermore, at an increased E:T ratio of 5:1, standard for CAR co-culture assays, CD4CAR NK-92 cells lysed 82% of Sézary syndrome cells from patient 1, and 82% of T-ALL cells from patient 2 (N=2) (Figures 3C and Supplementary Figure 1). Likewise, these data demonstrate a dose-dependent response and profound CD4CAR NK-92 cell anti-tumor activity in a cell line and patient sample setting for both adult and pediatric CD4^+^ T cell leukemias and lymphomas.

In order to verify the robustness of our *ex vivo* findings, we measured absolute numbers to determine the extent of CD4CAR NK cell cytotoxicity, in order to rule out the potential confounding factor of CD4CAR NK-92 cell expansion during the short 4 to 24h co-culture (Supplementary Figure 4). Absolute number analysis for target cell lines, patient samples, and negative control, independently verified the above tumorlysis data percentages obtained by relative frequency. This confirmation is consistent with our previously published work and other unpublished data. Additionally, for a short incubation time of 4h, CD4CAR NK cells already lyse 80% of KARPAS-299 cells at an E:T of 5:1, a timeframe for which effector cells would not have time to meaningfully expand (Supplementary Figure 3C), consistent with obtained absolute number data (Supplementary Figure 4).

### CD4CAR NK-92 cells specifically lyse CD4-expressing tumor cell lines in dose dependent manner

**Figure 4:**
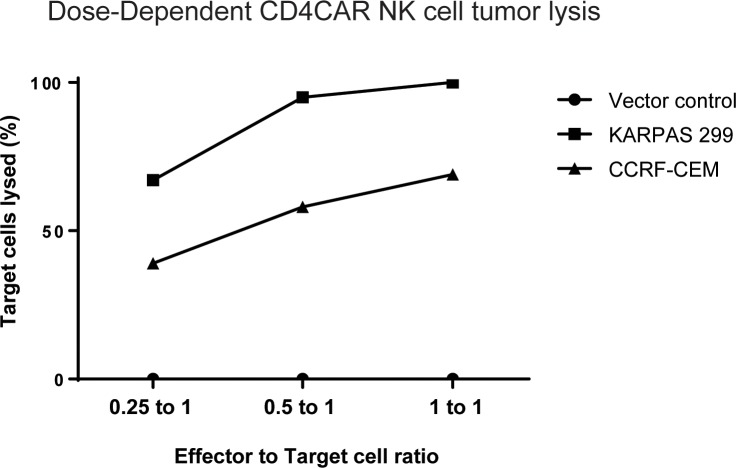
Co-culture specificity and dose response killing curve CD4CAR NK-92 cells lyse CD4-expressing leukemic cell lines in a dose-dependent and specific manner. CD4CAR NK and vector control cells were incubated with an equal ratio of CFSE-stained “on-target” (KARPAS-299 or CCRF-CEM) cells and CMTMR-stained “off target” MOLT4 cells at 0.25 to 1, 0.5 to 2, and 1 to 1 effector to target ratios. After 24 hours, 7-AAD dye was added and remaining live cells were analyzed by flow cytometry. Percent killing of target cells was measured by comparing CD4^+^ KARPAS-299 or CCRF-CEM cell survival in CD4CAR NK-92 cell co-cultures relative to survival in vector control NK-92 cell co-cultures.

CD4CAR NK-92 cells specifically lyse CD4^+^ KARPAS-299 and CCRF-CEM leukemic cell lines *ex vivo* in a dose-dependent manner at effector:target (E:T) ratios of 1:4, 1:2, and 1:1 (Figure 4). For each co-culture, CD4CAR or vector control NK-92 effector cells were incubated with tumor cells that were comprised of equal numbers of on-target CD4^+^ cells, CFSE-stained KARPAS-299 or CFSE-stained CCRF-CEM, and “off-target” CMTMR-stained CD4^-^, CD5^+^ MOLT4 acute lymphoblastic leukemia cells [45]. The MOLT4 cells were included to account for variation in the starting cell numbers and for spontaneous target cell death. After 24 hours, live cells were analyzed by flow cytometry. Percent lysis of target cells was measured by comparing CD4^+^ target cell survival in CD4CAR NK-92 co-culture to vector control NK-92 co-culture. KARPAS-299 cells were eliminated at rates of 67%, 95%, and 100%, at effector to target ratios of 0.25 to 1, 0.5 to 1, and 1 to 1, respectively (Figure 4), while, CCRF-CEM cells were eliminated at rates of 39%, 58%, and 69% respectively at the same E:T ratios (Figure 4). As expected, CD4CAR NK-92 cells did not lyse CMTMR-labeled MOLT4 cells, confirmed to be <5% CD4^+^ by flow cytometry analysis (Supplementary Figure 3A). Additional co-culture experiments confirmed that CD4CAR NK-92 cells did not lyse MOLT4 cells at 0h, 4h, 8h, and 24h (Supplementary Figure 3B), whereas CD4CAR NK-92 cells lysed KARPAS-299 cells as detected by flow cytometry as early as 4h (Supplementary Figure 3C). Combined, these data indicate that CD4CAR NK-92 cell anti-tumor cytotoxicity is dose-dependent, of rapid onset and is specific to CD4^+^ cells.

Additional co-culture studies were conducted using CD4^+^ T-cells isolated from cord blood. In these experiments, CD4CAR NK-92 cells completely depleted CD4^+^ T-cells at an effector:target ratio of 2:1 after 24 hours of co-culture, with remaining cells 0.0% CD4^+^. As expected, after CD4^+^ cord blood cell co-culture with corresponding vector control NK-92 cells (CD56^+^, CD4^-^), the CD4^+^ population remained largely intact (Figure 5A), further confirming specific and robust CD4CAR NK-92 mediated depletion of CD4^+^ populations in healthy tissue.

### CD4CAR NK-92 cells do not affect stem cell output in hematopoietic compartment

CFU (Colony-Forming-Unit) assay analysis revealed that CD4CAR NK-92 cells did not significantly affect the CD34+ cord blood stem cell output of the hematopoietic compartment. Hematopoietic compartment output was assessed by the presence of erythroid progenitors and granulocyte/macrophage progenitors at Day 0, determined by number of erythroid burst-forming units (BFU-E) and number of granulocyte/monocyte colony-forming units (CFU-GM) at Day 16 (Figure 5B). This finding is consistent with specific targeting of CD4, a mature T-cell marker, with limited impact on hematopoietic stem cells and early progenitors, and no evidence of lineage skewing, a measure of therapeutic safety.

### CD4CAR NK-92 cells exhibit significant anti-tumor activity *in vivo*

**Figure 6:**
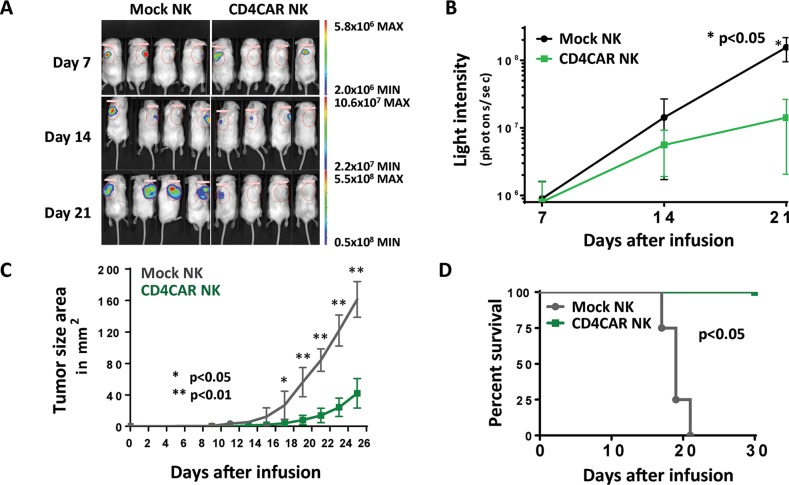
CD4CAR NK-92 cells demonstrate anti-leukemic effects *in vivo* NSG mice were sublethally irradiated and intradermally injected with luciferase-expressing KARPAS-299 cells (Day 0) to induce measurable tumor formation. On day 1 and every 5 days for a total of 6 courses, mice were intravenously injected with 5 × 10^6^ CD4CAR NK-92 cells or vector control NK-92 cells. **(A)** On days 7, 14, and 21, mice were injected subcutaneously with RediJect D-Luciferin and subjected to IVIS imaging. **(B)** Average light intensity measured for the CD4CAR NK-92 injected mice was compared to that of vector control NK-92 injected mice. **(C)** On day 1, and every other day after, tumor size area was measured and the average tumor size between the two groups was compared. **(D)** Percent survival of mice was measured and compared between the two groups.

In order to evaluate the *in vivo* anti-tumor activity of CD4CAR NK-92 cells, we developed a xenogeneic mouse model using NSG mice sublethally irradiated and intradermally injected with luciferase-expressing KARPAS-299 cells to induce measurable tumor formation. Subcutaneous injection of KARPAS-299 allows for modeling of difficult-to-access lymphoma nodules and provides accessible measurement of visible tumor growth. On day 1, 24 hours following KARPAS-299 cell injection, mice were intravenously injected with a one course dose consisting of 15 × 10^6^ CD4CAR or vector control NK-92 cells through the NK cell life span of around 2 weeks. On days 7, 14, and 21, mice were injected subcutaneously with RediJect D-Luciferin and underwent IVIS imaging to measure tumor burden (Figure 6A). Average light intensity measured for the CD4CAR NK-92 injected mice was compared to that of vector control NK injected mice (Figure 6B). By Day 21, the CD4CAR NK-92 injected mice had significantly less light intensity and therefore thus less tumor burden compared to vector control (p <0.01). On day 1, and every other day afterwards, tumor size area was measured and the average tumor size between the two groups was compared (Figure 6C). Unpaired student T test analysis revealed that the average tumor size of CD4CAR NK-92 injected mice was significantly smaller than that of vector control injected mice starting on day 17 (p <0.05) and continuing on days 19-25 (p <0.01). At the endpoint of the first course of NK cell life expectancy (after day 14), both groups were injected with 3 small subsequent booster doses of 5 × 10^6^ NK cells. We compared mouse survival across the two groups (Figure 6D). All of the CD4CAR NK-92 injected mice survived past day 30. However, percent survival of vector control NK-92 injected mice started to decrease on day 17 with no survival by day 23, determined by upper limit subcutaneous tumor size standards for mice. In summary, these *in vivo* data indicate that CD4CAR NK-92 cells significantly reduce tumor burden and prolong survival in KARPAS-299-injected NSG mice. Finally, it is important to note that the xenograft *in vivo* murine model does not fully recapitulate the human microenvironment for CD4CAR NK-92 cells, which are not supported by human cytokines and other factors, as they would be in a clinical setting. As a result, the xenograft model potentially underestimates actual CD4CAR NK-92 cell anti-tumor potency.

## DISCUSSION

PTCLs are aggressive lymphomas with no well-defined treatment standard, and almost no effective curative options. CAR immunotherapy has largely focused on targeting B-cell malignancies, with no reports of CAR-modified NK cells targeting PTCLs or T-cell malignancies. In our study, we provide novel targeting of CD4 using CAR-modified NK cells. Indeed, CD4CAR NK-92 cells specifically and effectively target CD4-expressing leukemia and lymphomas, as a proof of concept for allogeneic or autologous NK cell-based CAR therapy for CD4^+^ PTCLs. CD4CAR NK-92 cells reliably lysed 100% of aggressive KARPAS-299 cell lines at a low E:T 2:1 ratio, and lysed patient samples at >80% at an E:T ratio of 5:1 *ex vivo.* At corresponding E:T ratios and adjusting for cytotoxicity assay culture conditions, CD4CAR NK-92 tumorlysis compares favorably to pre-clinical data supporting the first anti-CD19 CAR clinical trial [46]. Although many immunotherapy studies require E:T ratios of 5:1 or more to demonstrate efficacy in the standard cytotoxicity assay, we demonstrate robust tumorlysis at both 5:1 and the reduced 2:1 ratios, the latter for added rigor. Notably, CD4CAR NK-92 cells eradicated KARPAS-299 tumor growth *in vivo*.

CAR NK cells are capable of serially killing tumor cells, and thus are an attractive immunotherapy strategy for PTCLs [21]. As a mature marker restricted to the hematopoietic compartment, CD4 is an attractive target that restricts on-target, off-tumor effects to healthy CD4^+^ cells in the hematopoietic compartment [47]. NK cells are unique effector cells in that they possess innate tumor-associated antigen independent cytotoxicity via multiple natural cytotoxicity receptors [48]. Additionally, they have the capacity for antibody-dependent cell-mediated cytotoxicity as they express the IgG Fc fragment, low affinity III receptor (FcRYIII). Compared to CAR T-cells, CAR NK cells have the advantage of targeting tumor cells via multiple mechanisms, with a less pro-inflammatory cytokine profile. However, primary blood NK cells present significant biological and logistical challenges for cancer therapy as they may be blocked by elements of innate immune response and compromise of immunologic function by underlying disease. Furthermore, the difficulty of culturing primary NK cells presents obstacles in generating enough therapeutic units for sufficient dosing. To this end, there has been great interest in generating expandable allogeneic NK cell lines, although to date, only the NK-92 cell line possesses a degree of consistent cytotoxic activity. Therefore, NK-92 cell lines have seen both significant preclinical work [26–39] as well as input into various clinical trials [21, 31–32]. Indeed, anti-CD19 CAR NK cells are being studied in patients with B-cell ALL (NCT00995137 and NCT01974479).

Clinically, a central concern is the potentially deleterious effect of CD4 T cell aplasia with regards to opportunistic infections, viral reactivation, and post-transplant lymphoproliferative disorder. For this reason, CD4CAR NK transient antitumor activity may be preferred, with reversible CD4 aplasia. The antigen recognition domain of the CD4 CAR is derived from ibalizumab, a commercial anti-CD4 antibody. Multiple clinical studies using monoclonal antibody-based therapies such as ibalizumab targeting CD4 have shown that transient ablation of CD4-expressing cells is well-tolerated in patients with T-cell lymphoma without evidence of irreversible immunosuppression or other long-term adverse events [14, 15, 17]. We expect NK CD4 CAR cells will have a short lifespan of approximately 2 weeks, in stark contrast to T cells which can persist and engraft for years [20]. Thus, it is expected that NK cells would be exhausted shortly after destroying cancer cells, and as a result, CAR-modified NK cells is likely to have a lower risk of long-term toxicity. Future incorporation of an inducible suicide switch could provide added therapeutic safety by ensuring a transient effect.

Alternatively, the argument that NK cells are too short lived to provide persistent responses for remission can be ameliorated through dosing in multiple intervals or, more likely, incorporation of cytokines or CAR constructs modified with cytokine production to allow for longer lasting NK cells. In fact, recent published work using CAR constructs incorporating IL15 show prolonged persistence of NK CAR cells, without negative effects on overall cytotoxicity and observations of anti-tumor activity [49]. These observations concur with our own experiments using IL15 based ‘armor’ to enhance immune cell persistency and efficacy (unpublished data).

In addition to the need for reversible CD4 aplasia, a secondary clinical concern is targeting of CD4^+^ T cells in the thymus. The vast majority of patients with untreatable CD4^+^ malignancies are adults who would not be affected by CD4CAR-mediated reduction in thymus volume and function. Even for the pediatric population, there is evidence that thymus reconstitution would occur following discontinuation of CD4CAR therapy [50–52].

CD4CAR NK therapy would be a particularly attractive therapeutic option for CD4^+^ malignancies in patients with minimal residual disease that is resistant to standard chemotherapy. In these cases, CD4CAR NK therapy could be used to specifically target and eliminate cancer cells before falling out of circulation. Furthermore, there may be no subsequent need for hematopoietic stem cell transplant/stem cell rescue following CD4CAR NK therapy, as hematopoietic stem cells do not uniformly express CD4 and myeloablation would not be expected, as supported by our CFU data.

CD4CAR NK cells could also be potentially used as a bridge to hematopoietic stem cell transplant in candidates who do not meet criteria for transplant due to a small percentage of residual blasts following standard chemotherapy treatment. These potential clinical indications for CD4CAR are particularly significant given the markedly poor prognosis associated with T-cell malignancies [4].

Because the CD4CAR construct scFv is derived from the humanized monoclonal antibody ibalizumab (Hu5A8 or TNX-355), we propose that the *in vivo* specificity of the CD4CAR should be similar, if not nearly identical, to that of ibalizumab. Clinical studies to date utilizing ibalizumab have already characterized its efficacy and safety profile in patients with HIV [40]. Additionally, to our knowledge this is the only anti-CD4 CAR studied for hematologic malignancies to date. Thus, data from previous clinical trials combined with our pre-clinical efficacy and safety data suggests that CD4CAR NK cells have appropriate specificity for targeting CD4^+^ malignancies.

Used as a stand-in for NK cells in general, the NK-92 clonal cells used in this study are experimentally reliable. NK-92 cells pose a low tumorigenicity risk when irradiated and transfused in oncology patients [52]. Additionally, NK-92 cells are dependent on IL-2 for growth and cytotoxic activity, and therefore have high fidelity for endogenous NK cell function, allowing for experimental control of NK-92 expansion. Overall, NK-92 cell have been deemed safe and effective in oncologic clinical trials: irradiation of NK-92 cells halts cell division without diminishing cytotoxicity [31, 32, 52, 53], and therefore are an ideal NK model for our pre-clinical efficacy study.

Although the scope of our pre-clinical study is to demonstrate the feasibility and efficacy of redirecting NK cells against CD4^+^ T-cell malignancies, the future case for a favorable safety profile of this novel immunotherapy is supported by the relatively short half-life of NK effector cells, the anticipated transient nature of treatment-induced CD4^+^ lymphopenia, the absence of CD4 on hematopoietic stem cells/progenitors, and previous studies with anti-CD4 monoclonal antibodies. In a clinical setting, NK CAR therapies could be construed as a potential universal “off-the-shelf” option that does not require human leukocyte antigen (HLA) matching. Although human NK cells have a turnover time in blood of about 2 weeks [20], concerns regarding immunotherapy persistence or prolonged CD4^+^ cell aplasia can be addressed by the inclusion of an inducible suicide gene into the CAR-modified NK cells [21, 22]. Multiple dose administration of CD4CAR NK cells could be potentially used as a stand-alone therapy, without transplant, for the treatment of aggressive CD4^+^ PTCLs.

## MATERIALS AND METHODS

### Primary tumor cells and cell lines

Human leukemia cells were obtained from residual samples on a protocol approved by the Institutional Review Board of Stony Brook University. Cord blood cells were also obtained under protocol from donors at Stony Brook University Hospital. Written, informed consent was obtained from all donors. KARPAS-299, HL-60, CCRF-CEM, MOLT4 and NK-92 cell lines were obtained from ATCC (Manassas, VA). NK-92 cells were cultured in filtered NK cell media, defined as alpha-MEM without ribonucleosides and deoxyribonucleosides with 2mM L-glutamine, 1.5 g/L sodium bicarbonate, 12.5% heat-inactivated horse serum, 12.5% heat-inactivated FBS, 1X Pen/Strep, 0.2% inositol, 0.02% folic acid, and 50 uM beta-mercaptoethanol, supplemented with IL-2 (300 IU/mL), unless otherwise specified. KARPAS-299, CCRF-CEM, and MOLT4 cell lines were cultured in RPMI, 10% FBS, 1x Pen/Strep (Gibco, Waltham, MA, USA). HL-60 cells were cultured in IMDM, 10% FBS, 1x Pen/Strep (Gibco, Waltham, MA, USA).

### CAR construct generation

The CD4-specific CAR (pRSC.SFFV.CD4.3G) was designed to contain an intracellular CD28 domain upstream of 4-1BB and CD3zeta domains, defining the construct as a third-generation CAR.

### Lentivirus production and transduction

To produce viral supernatant, 293FT-cells were co-transfected with pMD2G and pSPAX viral packaging plasmids containing either pRSC.SFFV.CD4.3G or GFP lentiviral vector control, using Lipofectamine 2000 (Life Technologies, Carlsbad, CA) per manufacturer's protocol.

NK-92 cells were cultured for a minimum of 2 days in the presence of 300 IU/mL IL-2 prior to transduction with viral supernatant. Transfection and transduction procedures are further described in Supplemental Data.

### CAR detection on transduced NK-92 cells

In order to determine CAR expression, NK-92 cells were washed and suspended in FACs buffer (0.2% BSA in DPBS) 3 days after transduction. Normal goat IgG (Jackson Immunoresearch, West Grove, PA) was used to block nonspecific binding. Each NK cell sample was probed with Biotin-labeled polyclonal goat anti-mouse F(Ab’)^2^ (1:250, Jackson Immunoresearch, West Grove, PA) for 30 minutes at 4°C. Cells were washed once, and resuspended in FACs buffer. Cells were then stained with PE-labeled streptavidin (1:250, Jackson Immuno Research, West Grove, PA) for 30 minutes at 4°C. Cells were washed with FACs buffer, and resuspended in 2% formalin. Flow cytometry was performed using a FACS Calibur instrument (Becton Dickinson, Franklin Lakes, NJ), and results were analyzed using Kaluza software (Beckman Coulter, Brea, CA).

### Co-culture assays

CD4CAR or vector control NK-92 cells were incubated with CD4 expressing KARPAS-299 cells (anaplastic large T-cell lymphoma), HL-60 cells (acute promyelocytic leukemia), CCRF-CEM cells (T-cell acute lymphoblastic leukemia: T-ALL), CD4^+^ T-cells isolated from human cord blood, or CD4 expressing primary human leukemic cells (adult Sézary syndrome and pediatric T-ALL) at ratios of 2:1 and 5:1 (200,000 and 500,000 effector cells to 100,000 target cells, respectively) in 1 mL of NK-cell culture media, without IL-2. After 24 hours of co-culture, remaining live cells were harvested and stained with mouse anti-human CD56 and CD4 antibodies, and were incubated at 4°C for 30 minutes. CD56^+^ single positive denoted NK cells, and CD4^+^ single positive denoted target cells. All cells were washed with FACs buffer, suspended in 2% formalin, and analyzed by flow cytometry. Full list of antibodies can be found in Supplementary Table 1.

### Cytotoxicity assay

CD4CAR or vector control NK-92 cells were incubated with an 50:50 mix of on-target cells (CFSE-stained KARPAS-299 cells and CMTMR-stained CCRF-CEM cells) and off-target CMTR-labeled MOLT4 cells at effector: target ratios of 1:1, 1:2, and 1:4 ratios in 1 mL of NK-cell culture media, without IL-2 [44]. After 24 hours, cells were stained with 7-AAD (BioLegend, San Diego, CA), washed with FACS buffer, and live 7-AAD negative cells were analyzed by flow cytometry.

### Colony forming unit (CFU) assay

CD4CAR NK-92 cells were incubated at co-culture effector: target ratios of 2:1 and 5:1 respectively with 500 CD34+ CB cells for 24 hours in NK cell media supplemented with IL-2. Controls used were CD34+ cells alone, and non-transduced NK-92 cells co-cultured at 2:1 and 5:1 effector:target ratios with CD34+ CB cells. Hematopoietic compartment output was assessed via formation of erythroid burst-forming units (BFU-E) and number of granulocyte/monocyte colony-forming units (CFU-GM) at Day 16. CFU statistical analysis was performed via 2-way ANOVA with alpha set at 0.05.

### Xenogeneic mouse model

Male 12-week-old NSG mice (NOD.Cg-Prkdcsid Il2rgtm1Wjl/SzJ) were purchased from the Jackson Laboratory (Bar Harbor, ME) and used under a Stony Brook University IACUC-approved protocol. NSG mice were irradiated with a sublethal (2.5 Gy) dose of gamma irradiation. Twenty-four hours later, mice were intradermally injected with 0.5 x10^6^ KARPAS-299 cells that had been stably transduced to express luciferase, in order to cause a measurable subcutaneous tumor to form. On day 1, forty-eight hours following KARPAS-299 cell injection, mice were intravenously injected via tail vein with a one course dose of 15 × 10^6^ CD4CAR or vector control NK-92 cells (N=4 per group). Tumor size area was measured every other day. After day 14 (NK lifespan endpoint), mice were injected with 3 subsequent low doses of 5 × 10^6^ NK cells through day 30. On days 7, 14, and 21 following KARPAS-299 cell injection, mice were injected subcutaneously with 100 μL RediJect D-Luciferin (Perkin Elmer, Waltham, MA) and subjected to IVIS imaging (PerkinElmer, Waltham, MA). Images were analyzed using Caliper Life Sciences software (PerkinElmer, Waltham, MA).

### Statistics

Xenogeneic model sample sizes were estimated using 2-sample, 2-sided equality power analysis (90% power and <5% significance). Unpaired Student T tests were used to determine significance of tumor size area and light intensity. Survival curves were constructed using the Kaplan-Meier method and statistical analyses of survival was performed using a log-rank (Mantel-Cox) test with P <0.05 considered significant. Statistical analyses were performed using GraphPad Prism 6 software. Variance was determined to be similar between the treatment and control group prior to unpaired student-test.

## SUPPLEMENTARY MATERIALS FIGURES AND TABLES


