# Supplementary material for: Targeting T-cell malignancies using anti-CD4 CAR NK-92 cells
Source: Oncotarget. 2017 Nov 22;8(68):112783–96. doi: 10.18632/oncotarget.22626 (PMC5762550; doi:10.18632/oncotarget.22626)
Supplement: Supplementary file 1 [file oncotarget-08-112783-s001.pdf]

Targeting T-cell malignancies using anti-CD4 CAR NK-92 cells

SUPPLEMENTARY MATERIALS

Detailed lentivirus production and transduction of NK-92 cells

To produce viral supernatant, 293FT-cells were co-transfected with pMD2G and pSPAX viral packaging plasmids containing either pRSC.SFFV.CD4.3G or GFP lentiviral vector control, using Lipofectamine 2000 (Life Technologies, Carlsbad, CA) according to the manufacturer's protocol, and incubated for 6 hours. Cells were then washed and suspended in DMEM with 10% FBS, sodium butyrate, sodium pyruvate, and HEPES (20mM) (all Gibco, Waltham, MA, USA). Viral supernatant was collected 24 and 48 hours after transfection, cleared of cellular debris via centrifugation and filtration (0.45 uM), aliquoted, and flash frozen in liquid nitrogen for storage at -80°C.

To confirm virus production, 293-FT cells were harvested 48 hours after transfection, lysed in 1 mL RIPA buffer with deoxycholate and protease inhibitor cocktail [63], and 10 uL sample was electrophoresed on a 10% PAGE-SDS gel, and transferred to Immobilon FL (0.45 uM) membrane using the wet cell method. Milk (5%) in TBS/Tween was used to block blots. Blots were probed with anti-CD247/CD3z (Thermo Fisher Holtsvile, NY) at 1:500 overnight, washed 4 times with TBS/Tween, and probed with anti-goat IgG, HRP-conjugated antibody (Thermo Fisher) at 1:5000 for 2 hours. Following additional washes, HRP substrate (HyGlow, Denville, Holliston, MA) was added to the membrane and the membrane was exposed to autoradiographic film.

NK-92 cells (ATCC; Manassas, VA) were cultured for 2 days in the presence of 300 IU/mL IL-2 (Miltenyi Biotec, Bergisch, Gladbach, Germany). A non-tissue culture treated 6-well plate was coated with RetroNectin (Clontech, Mountain View, CA) at 15 ug/mL in DPBS for 2 hours at room temperature or overnight at 4 °C. Wells were blocked with 2% BSA in PBS for 30 minutes at room temperature, then washed once with PBS. Viral supernatant (CD4CAR or GFP vector control lentivirus) was diluted 1:1 with DMEM containing 10% FBS and added to the washed wells by centrifugation at 2000 g for 2 hours at 32 °C. Wells were washed once with NK cell media, and NK-92 cells were added, 4 mL per well at 0.5 x 10^6 cells/mL, with IL-2 (300 IU/mL. Plates were centrifuged at 1000 g for 10 minutes and incubated overnight at 37 °C in the presence of 5% CO_2. The following morning, a second transduction, identical to the first, was carried out. The morning after that, cells were transferred to a fresh non-coated 6-well plate in NK cell media with IL-2 (300 IU/mL), cells were sorted for $CD4CAR^+$ NK-92 cells, and subsequently incubated as above for a total of 7 days from transduction.

Colony formation unit assays

CFU assays were conducted in 4-7 replicates per set in 35mm dishes in MethoCult H4435 Enriched (Stem Cell Technologies, Vancouver, Canada), optimized for CD34+ purified cord blood. CFU statistical analysis was performed via 2-way ANOVA with alpha set at 0.05.

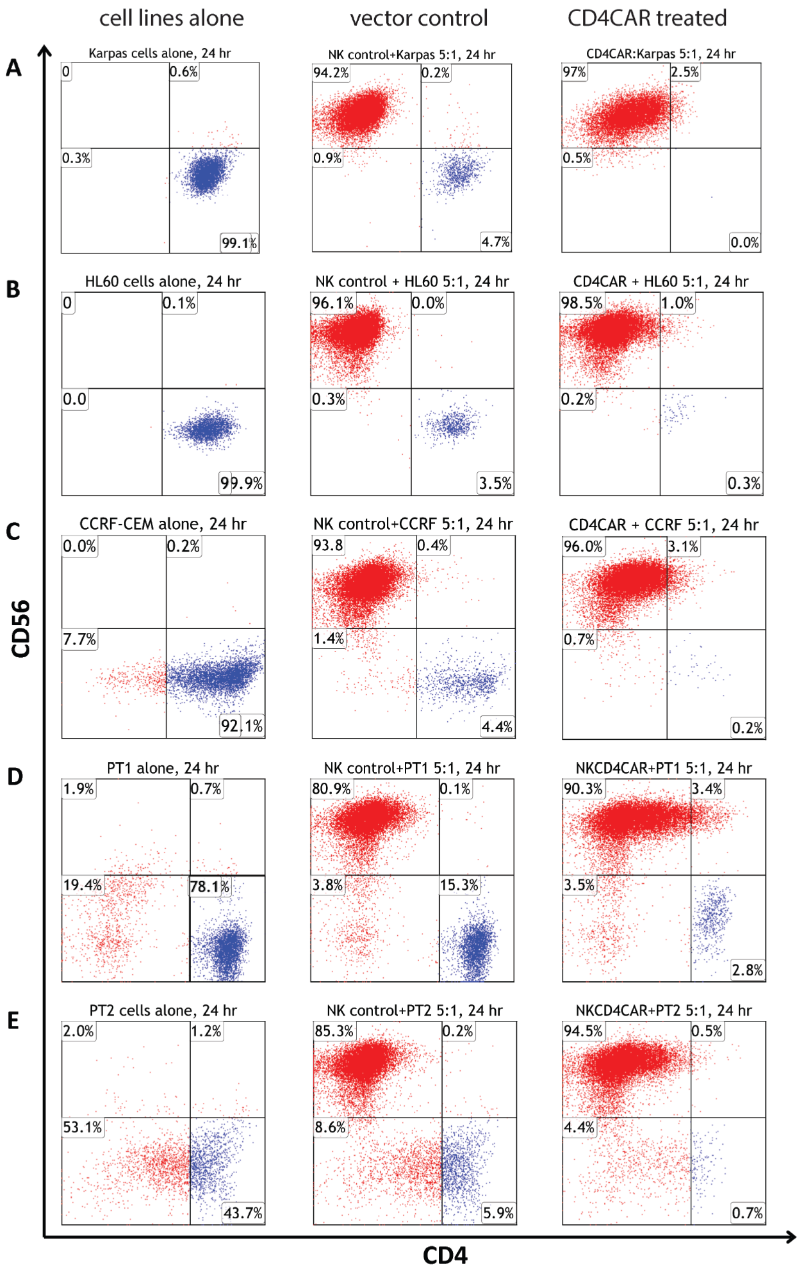

Supplementary Figure 1: CD4CAR NK-92 cells ablate CD4+ leukemia and lymphoma cells in ex vivo co-culture assays. All co-culture assays shown were performed at an effector to target ratio of 5:1 for 24 hours, after which, cells were stained with mouse anti-human CD56 and CD4 antibodies. Each assay consists of NK-92 cells transduced with either vector control (center) or CD4CAR (right) lentiviral supernatant and incubated with target cells, as well as target cells incubated alone as a control (left). **(A)** CD4CAR NK-92 cells eliminated Karpas 299 leukemic T-cells, **(B)** HL-60 T-cells, and **(C)** CCRF-CEM cells. (**D**) CD4CAR NK-92 cells eliminated primary T-cell leukemia cells from patients with CD4 expressing T-cell leukemia/ Sézary syndrome and **(E)** CD4-expressing pediatric T-cell ALL.

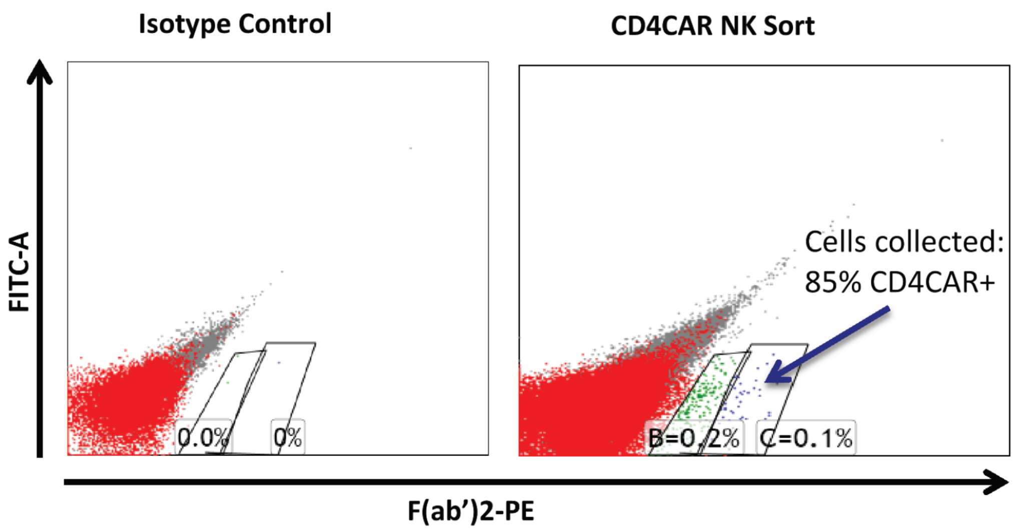

Supplementary Figure 2: NK-92 cells were transduced with either vector control or CDCAR lentiviral supernatant, or cultured for non-transduced control. After 7 days of incubation, cells were harvested and analyzed by flow cytometry with Biotin-labeled goat anti-mouse F(Ab')2 followed by streptavidin-PE. NK-92 cells were >85% CD4CAR^{+} after sorting.

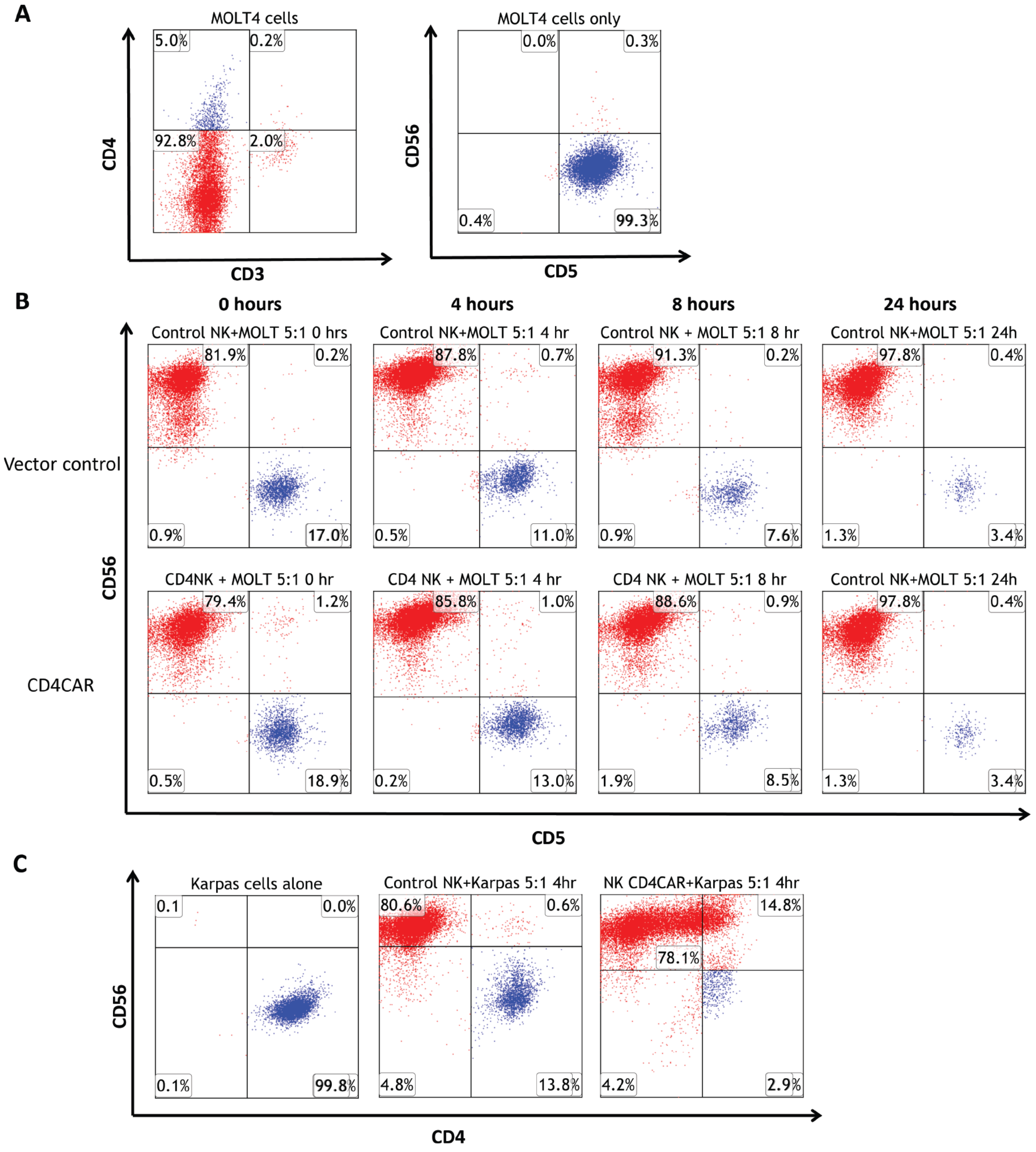

Supplementary Figure 3: CD4CAR NK-92 cells did not lyse CD4-, CD5+ MOLT4 negative control. **(A)** MOLT4 cell immunophenotype was confirmed to be almost all $CD4^-$ and $CD5^+$. **(B)** CD4CAR NK-92 cells did not lyse MOLT4 cells at a 5:1 effector to target ratio at 0h, 4h, 8h, and 24h (lower panel) as assessed by comparison to vector control NK cell tumorlysis (upper panel). **(C)** Anti-CD4 CDCAR NK-92 antitumor activity was confirmed at 4h with a $CD4^+$ Karpas 299 positive control at an 5:1 E:T ratio.

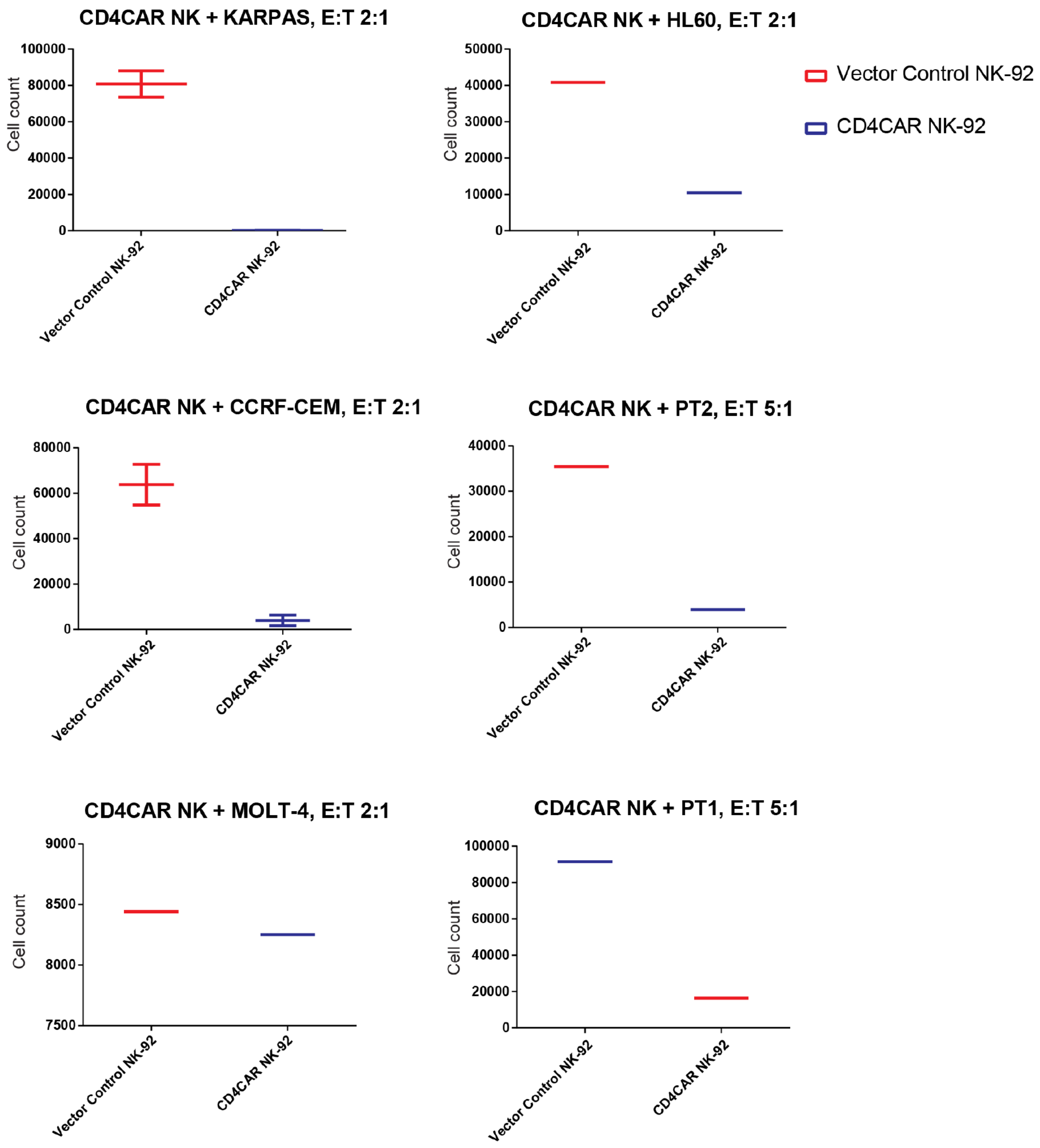

Supplementary Figure 4: Corresponding absolute cell numbers for ex vivo cytotoxicity assays for given effector: target ratios (E:T).

Supplementary Table 1: Flow cytometry, Western blot reagents

NAME	VENDOR	HOST SPECIES	CATALOG #	CLONE#	DILUTION	USE
CD247/CD3z	Thermo Fisher	mouse	MA5-15608	4B10	1:500	Western Blot
Goat anti-mouse HRP	Abnova	goat	PAB10746	n/a	1:2000	Western Blot
Goat anti-mouse F(Ab')2, biotin	Jackson	goat	115-066-072	n/a	1:250	F(Ab')2 detection/ Flow cytometry
R-Phycoerythrin Streptavidin	Jackson	n/a	016-110-084	n/a	1:250	Secondary for F(Ab')2 detection/ Flow cytometry
Anti-human CD4-APC	BD Biosciences	mouse	340443	SK3	1:33	Flow cytometry
Anti-human CD5-APC	Tonbo Biosciences	mouse	20-0059-T100	UCHT2	1:20	Flow cytometry
Anti-human CD56-PE	Tonbo Biosciences	mouse	50-0564-T100	NCAM, MY31	1:20	Flow cytometry
CMTMR	Life Technologies	n/a	C2927	n/a	5 µM	Flow cytometry
CFSE	Life Technologies	n/a	C34554	n/a	0.5 µM	Flow cytometry
